# A protocol for the formative evaluation of the implementation of patient-reported outcome measures in child and adolescent mental health services as part of a learning health system

**DOI:** 10.1186/s12961-024-01174-y

**Published:** 2024-07-15

**Authors:** Erin McCabe, Michele Dyson, Deborah McNeil, Whitney Hindmarch, Iliana Ortega, Paul D. Arnold, Gina Dimitropoulos, Ryan Clements, Maria J. Santana, Jennifer D. Zwicker

**Affiliations:** 1 School of Public Policy and Department of Pediatrics, University of Calgary, Calgary, Canada; 2 Provincial Addictions and Mental Health, Alberta Health Services, Edmonton, Canada; 3 Maternal Newborn Child and Youth Strategic Clinical Network, Alberta Health Services, Edmonton, AB Canada; 4 Department of Radiology, University of Calgary, Calgary, Canada; 5 Department of Psychiatry and Mathison Centre for Mental Health Research and Education, Hotchkiss Brain Institute, University of Calgary, Calgary, Canada; 6 Faculty of Social Work, University of Calgary, Calgary, Canada; 7 Alberta Health Services Calgary Zone, Calgary, Canada; 8 Department of Pediatrics and Community Health Sciences, University of Calgary, Calgary, Canada; 9 School of Public Policy and Faculty of Kinesiology, University of Calgary, Calgary, Canada; 10 Department of Community Health Sciences and Faculty of Nursing, University of Calgary, Calgary, Canada

**Keywords:** Learning health system, Mental health services, Measurement-based care, Patient-reported outcome measures, Mental illness, Pediatric mental health, Adolescent mental health, Value-based health care, Implementation science

## Abstract

**Background:**

Mental health conditions affect one in seven young people and research suggests that current mental health services are not meeting the needs of most children and youth. Learning health systems are an approach to enhancing services through rapid, routinized cycles of continuous learning and improvement. Patient-reported outcome measures provide a key data source for learning health systems. They have also been shown to improve outcomes for patients when integrated into routine clinical care. However, implementing these measures into health systems is a challenging process. This paper describes a protocol for a formative evaluation of the implementation of patient-reported measures in a newly operational child and adolescent mental health centre in Calgary, Canada. The purpose is to optimize the collection and use of patient-reported outcome measures. Our specific objectives are to assess the implementation progress, identify barriers and facilitators to implementation, and explore patient, caregivers and clinician experiences of using these measures in routine clinical care.

**Methods:**

This study is a mixed-methods, formative evaluation using the Consolidated Framework for Implementation Research. Participants include patients and caregivers who have used the centre’s services, as well as leadership, clinical and support staff at the centre. Focus groups and semi-structured interviews will be conducted to assess barriers and facilitators to the implementation and sustainability of the use of patient-reported outcome measures, as well as individuals’ experiences with using these measures within clinical care. The data generated by the patient-reported measures over the first five months of the centre’s operation will be analyzed to understand implementation progress, as well as validity of the chosen measures for the centres’ population.

**Discussion:**

The findings of this evaluation will help to identify and address the factors that are affecting the successful implementation of patient-reported measures at the centre. They will inform the co-design of strategies to improve implementation with key stakeholders, which include patients, clinical staff, and leadership at the centre. To our knowledge, this is the first study of the implementation of patient-reported outcome measures in child and adolescent mental health services and our findings can be used to enhance future implementation efforts in similar settings.

**Supplementary Information:**

The online version contains supplementary material available at 10.1186/s12961-024-01174-y.

## Background

It is estimated that one in seven young people experience a mental illness and that about 70% of mental health challenges have their onset before the age of 18 [1, 2]. Anxiety disorders are the most common of these disorders affecting five percent of Canadian children aged 5–17 years old while 2.1% reported a mood disorder in 2019 [3]. Mental illness is one of the leading causes of disability among adolescents [1]. Despite the burden of mental health conditions in children and adolescents, data suggests that current mental health services are not meeting the needs of a majority of these children and youth. Only 44.2% of children experiencing a mental health condition received any services, revealing a large gap in services for children and youth mental health [4]. In Alberta, a Western Canadian province, the rates of emergency department visits for pediatric mental health concerns increased 35% between 2010 and 2020 [5], likely the result of a lack of availability of services for urgent mental health needs in the community [6]. The COVID-19 pandemic has further amplified the critical need for adequate mental health supports and services for children and youth [7, 8].

To address the needs of children and youth in Alberta, a new centre for children and adolescents with urgent mental health concerns, called “The Summit: Marian & Jim Sinneave Centre for Youth Resilience” (herein, The Summit) is opening in March 2023 in Calgary, Canada. The City of Calgary is located in the province of Alberta and is Canada’s third largest municipality, serving 1.3 million people [9]. The Summit will support children and youth (0–18 years old) with acute mental health concerns in a community setting. The centre will be operated by Alberta Health Services (AHS), the province-wide, publicly-funded health system in Alberta, Canada. The Summit has an integrated research program in partnership with the University of Calgary. This close partnership between academic researchers and the health system is supporting the development of The Summit as a learning health system (LHS).

As defined by Menear et al. (2019), an LHS is a “*dynamic health ecosystem where scientific, social, technological, policy, legal and ethical dimensions are synergistically aligned to enable cycles of continuous learning and improvement to be routinised and embedded across the system, thus enhancing value through an optimised balance of impacts on patient and provider experience, population health and health system costs*.” AHS is facing rising health care costs and is therefore moving towards a model of Value-Based Health Care (VBHC), where the purpoted aim is to offer patients the best possible outcomes weighed against the cost of providing services [10, 11]. The implementation of a LHS is seen as a pathway towards operationalizing VBHC and thus, establishing a LHS has become a priority for AHS [12].

Foundational to a LHS is the rapid executing of learning cycles, where data are generated within clinical practice and transformed to knowledge and that knowledge is rapidly applied back into clinical practice [11]. A key source of data in a learning health system is patient-reported outcomes (PROs), gathered with patient-reported outcome measures (PROMs). PROMs are validated questionnaires filled out by the patient about their health status, quality of life, or well-being [13]. PROMs provide valuable data about outcomes from the patient’s perspective that can be used within an LHS to answer questions regarding treatment effectiveness, disease trajectories, and the value of care provided [14]. PROMs also provide valuable information for the clinician and patient’s own learning, for example, whether a particular treatment is having the intended effect on a patient’s symptoms. The use of PROMs in routine clinical care can improve communication between patient and clinicians, enhance share-decision making, self-management and patient outcomes [15, 16].

Despite the value of PROMs within a health system, their successful implementation into routine clinical care is complex [17, 18]. It requires a significant investment of time and resources, as well as the development of a supportive information technology infrastructure [17, 19]. Commonly cited challenges to their implementation include finding appropriate PROMs (i.e., relevant to patients’ needs, feasible to use, and adding value to the clinical encounter), designing PROMs data collection processes around clinical workflows, getting buy-in from clinical staff, and creating a culture that is supportive of learning and quality improvement [17]. In child and youth mental health care, there are additional challenges, including finding PROMs that have been validated for their use in children and youth, developing data collection platforms and workflows that allow the collection of responses from both parents and children [20, 21]. There are also additional ethical questions to navigate, such as whether the youth, one caregiver, both parents or all should be responding to PROMs, whose responses will be more strongly weighted, whether a parent should respond to a PROM about their child without their child’s consent, and at what age a child has the right to keep their PROMs data private from their caregivers [21–23].

PROMs will be embedded into routine clinical care processes at The Summit when it opens. At the initial stages, the focus on PROMs will be their use as an intervention to enhance learning for the patient and clinician about the patients’ function and responses to treatment. PROMs data used for learning at the meso and macro levels will begin once sufficient data are collected for planned research projects and quality improvement, although the success of these activities will depend on the quality of PROMs data that is collected. Given the complex interactions between technical, organizational, personal and familial factors that may impact how effectively PROMs are collected and used for individual and family clinical care, it is crucial to assess and address unidentified or unresolved barriers to effective implementation in order to be successful in the implementation of PROMs at The Summit. The assessment will inform refinements to the components of the PROMs intervention and the implementation strategies, optimizing PROMs data collection and clinical use at the centre.

### Purpose

This paper describes a protocol for the formative evaluation of the implementation of PROMs for clinical care within The Summit. The purpose is to optimize the collection and use of PROMs. The evaluation objectives are to:

1. Assess the implementation progress to date (penetration, fidelity, appropriateness, and adoption)
2. Identify barriers and facilitators to implementation and sustainability of PROMs at The Summit
3. Explore patient, caregivers and clinician experiences of PROMs in clinical care

### Context

The Summit is a stand-alone facility which aims to serve children and youth ages 0–18 years old with emerging or worsening mental health concerns. It will have three main services: (1) a walk-in service for urgent mental health concerns; (2) a day program to support youth transitioning from a hospital admission back to their home; and (3) intensive community treatment services, designed to prevent the need for hospitalization, which provides youth with escalating mental health concerns with short-term, intensive therapy, consisting of group, family and individual therapy, and psychiatry services.

In partnership with researchers at the University of Calgary, The Summit has an embedded, patient-oriented research program with three functions: (1) generating new knowledge from data collected from patients at The Summit; (2) testing new interventions for children and youth with depression and anxiety; and (3) implementing and evaluating effectiveness of interventions that are not widely available to children and youth in Calgary. Additionally, the socioeconomic (i.e., cost-effectiveness and policy) impact of the interventions offered will be assessed.

### Intervention description

Measurement-Based Care (MBC) is a commonly used term to describe the routine clinical use of PROMs in mental health services [22]. It is accepted that MBC consists of three main steps: (1) administering a symptom, outcome, or process measure (i.e., PROM) ideally before a clinical encounter; (2) clinician and patient review of PROM data; (3) collaborative re-evaluation of the treatment plan informed by data [24].

MBC will be a standard part of care at The Summit. Three PROMs will be administered across all services at the Centre, the acute version of the Pediatric Quality of Life Inventory Generic Core Scales (PedsQL), the Revised Children’s Anxiety and Depression Scale (RCADS-25), and Columbia-Suicide Severity Rating Scale (C-SSRS). These measures were chosen based on the International Consortium for Health Outcomes Measurement’s Set of Patient-Centered Outcome Measures for Children and Young People with Anxiety and Depression, including OCD and PTSD, informed by the PROMs available within the provincial electronic health record (EHR), and in collaboration with patients, caregivers, and researchers [25].

The acute version of the PedsQL is a 23-item questionnaire with a 7-day recall period. It has 4 scales (physical, emotional, social, school functioning) and three summary scores (Physical Health Score, Psychosocial Health Score and Total Scale Score) [26]. It has self-report forms for children in 3 different age groups and parent-report forms for 4 different age groups [26]. RCADS-25 is a 25-item questionnaire for children and youth ages 8–18 with 2 subscales (anxiety and depression) and a total scale score which can be converted to norm-based T scores [27]. It has self-report and parent-report forms. The C-SSRS is a 7-item self-report questionnaire for youth ages 10 and older, which classifies individuals as high, moderate or low risk for suicide [28]. The RCADS-25 and C-SSRS have demonstrated good reliability and evidence of their validity in pediatric mental health populations [27, 28]. The reliability and validity of the PedsQL in this population has not been reported, however in the general population it performs well and is widely used within Alberta’s pediatric health system [29].

**Fig. 1:**
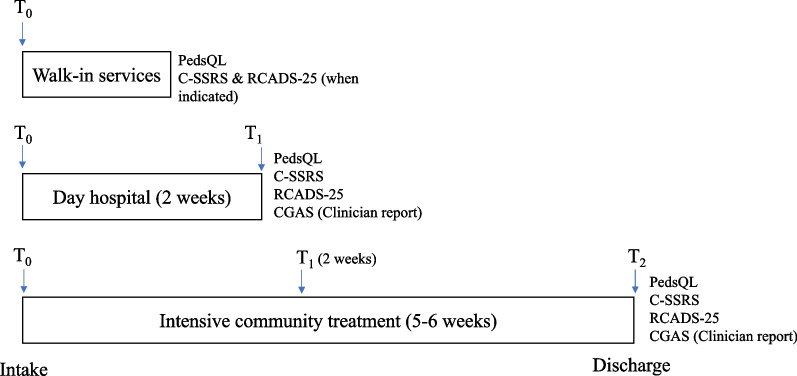
Patient-reported outcome measures in the three services at The Summit

Figure 1 illustrates PROM data collection timepoints for each service. For walk-in services, PedsQL will be administered routinely at intake, with the intake process flagging whether RCADS and C-SSRS need to be administered. For the 2-week day hospital program, all three PROMs will be administered at intake and at discharge. For intensive community treatment services, the three PROMs will be administered at intake, midpoint of treatment (after 2 weeks) and at discharge. In each case, the PROMs will be automatically scored and represented graphically (along with prior PROM scores) in a downloadable summary document which also contains patients’ responses to each item. The summary document will be imported into the patients’ EHR by the intake coordinator for the service. During the clinical encounter, the clinician will access patient scores in the EHR and review them with the patient. Together they will discuss patient concerns highlighted by the PROM and re-evaluate the treatment plan. Whenever possible, PROMs will be administered to both patients and caregivers. A clinician-reported measure, the Children’s Global Assessment Scale (CGAS), will be also be collected at each PROM collection timepoint in the day program and intensive community treatment services, as it is part of the standard of care for children and youth mental health services in Alberta [30].

### Implementation process

The implementation of PROMs at The Summit was guided by the Quality Implementation Framework (QIF) [31]. The QIF is an implementation process model which was designed to guide the “how-to” processes of implemenation [31]. It has 14 steps divided into four phases: (1) Initial considerations regarding the host setting, (2) Creating a structure for implementation, (3) Ongoing structure once implementation begins, (4) Improving future applications [31].

We formed a MBC working group consisting of operational leadership and academic partners (clinical and health systems researchers) 18 months prior to the opening of The Summit. The working group assessed the context; adapted the MBC intervention to The Summit; developed the information technology structure to collect, use and record PROMs in the patient EHR; and designed the implementation approach. The implementation strategies were developed based on existing PROMs implementation guidelines [24, 25, 32, 33]. We engaged youth and caregivers through The Summit’s advisory councils in designing the PROMs processes, selecting the PROMs to use and informing the implementation strategies.

Once an opening date for The Summit was finalized, an implementation team was created, consisting of two clinical leads from The Summit and 4 researchers. The implementation team finalized the PROMs clinical workflows and created a detailed plan for implementation. Once The Summit opens and PROMs collection begins, onsite technical and clinical support will be offered through the implementation team.

This formative evaluation is part of phase three of the QIF. We used the Implementation Research Logic Model (IRLM) to specify the core elements of the implementation design [34]. The IRLM for this project details the determinants, strategies, proposed mechanisms of change and outcomes for the MBC implementation project and is available as supplementary online material (Supplementary File 1).

## Methods

### Design

**Fig. 2:**
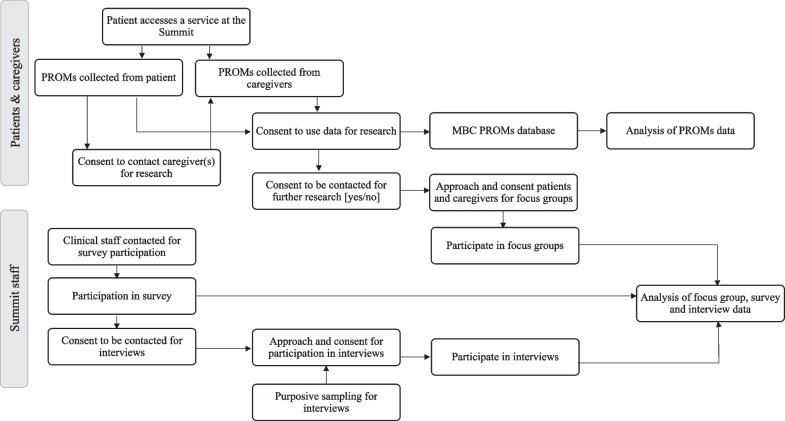
Study design flowchart

This study is a convergent mixed-methods, formative, implementation and progress-focused evaluation [35, 36]. This type of formative evaluation occurs during the implementation phase and focuses on identifying and resolving factors affecting implementation, as well as assessing progress towards the desired outcomes [35]. We will use the Consolidated Framework for Implementation Research (CFIR) to guide a comprehensive and systematic exploration of the factors affecting implementation of MBC at The Summit [37]. CFIR offers a menu of constructs that could influence implementation efforts, organized in five domains, some with subdomains: Innovation domain (i.e., MBC); Outer setting domain (i.e., the health system, academic institutions and community services surrounding The Summit); Inner setting domain (i.e., The Summit); Individuals domain (i.e., the roles and characteristics of The Summit staff, implementation team, and PROMs working group); Implementation process domain (i.e., implementation strategies, see Supplementary File 1) [37]. A detailed evaluation plan is included as supplementary material (Supplementary File 2). This study protocol was approved by The University of Calgary Research Ethics Boards (REB22-1137). Figure 2 illustrates the study design. A populated SPIRIT checklist has been included as supplementary material (Supplementary File 3) [38].

### Participants and recruitment

The MBC program will begin collecting PROMs data at The Summit opening (March 2023). Recruitment and data collection specific to this evaluation (surveys, interviews, focus groups) will begin three months after The Summit opens in order for staff and patients to develop a comprehensive experience of MBC. Sample sizes are based on feasibilty of recruitment and estimates to reach saturation point where no new information is forthcoming.

*Patients and caregivers* We will recruit 12–16 individuals ages 12–18 years old who accessed services at The Summit between March 2023 and August 2023 and have engaged in MBC (i.e., completed PROMs on 2 or more occasions). We will recruit 12–16 caregivers of patients (not necessarily paired with the patient participants) who accessed services at The Summit between March 2023 and August 2023 and who have been engaged in MBC (i.e., completed PROMs on two or more occasions).

A consent to be contacted for research purposes will be included within the PROMs data collection platform for each of the three services. Patients and caregivers who consent to be contacted for research will be approached for participation in focus groups. For day-program and intensive community treatment services, patients and caregivers will also be asked whether they consent for their PROMs data and clinical EHR data to be used for research. Patients accessing walk-in services may be in crisis, therefore it would not be appropriate to ask for consent to use data for research at the time of their visit, however they will be asked for consent to be contacted for research, and their consent to participate will be sought at a later date.

*The Summit staff* All Summit staff will be invited to participate in a survey through an email from The Summit staff members on the implementation team. For the interviews, staff will be invited to participate voluntarily through the survey, as well as purposively sampled to ensure a recruit a diverse sample representing healthcare professionals (e.g., social workers, psychologists, psychiatrists, psychiatric nurses), administrative support staff and leadership from across the three services.

### Data collection

All data will be stored on secure servers at the University of Calgary.

*Staff surveys (month 3–4)* The survey will be administered online with a mix of closed and open-ended questions about acceptibility, appropriateness, feasibility and fidelity, as well as general attitudes towards MBC at The Summit. The survey will be customized for each role (clinician, support staff, leadership).

*Staff interviews (months 4–6)* Semi-structured interviews will be conducted with 8–12 staff members to understand their attitudes towards and experiences with MBC at the Centre, and barriers and facilitators to implementation of MBC. Clinicians will also be asked about the appropriateness of the PROMs (i.e., PedsQL, RCADS-25, C-SSRS) and how scores are represented in the summary score sheet, and how they are using PROMs within clinical encounters (to assess fidelity). CFIR will inform the development of the interview guide.

*Patient and caregiver focus groups (months 4–6)* Focus groups will be held with patients and caregivers to understand their experiences with measurement-based care. Between 2–3 focus groups with 4–8 individuals will be held for both patients and caregivers, separately. The focus group interview guide will focus on usability of the platform, appropriateness of the PROMs, fidelity (are PROMs being discussed within their encounters), and perceptions of MBC as a part of their clinical care.

*Electronic Health Record Data (month 5)* De-identified patient data from the electronic health records of all patients attending The Summit will be acquired 5 months into operation. This will include the total number of patient encounters at the centre, the number of patients who have the PROMs summary document in their EHR (by service), and the number of patients who have the PROMs summary document at intake, midpoints of treatment and discharge.

*MBC PROMs database (month 5)* PROMs data collected through MBC from patients and caregivers consenting to data use for research will be matched with demographic and clinical data extracted from patients’ EHR (i.e., diagnosis, co-occurring diagnoses, sex and gender, language spoken at home, health care provider professional designation, number of episodes of care, types of therapeutic services accessed, clinician-reported measure scores, ethnicity including Indigeneity). Data will be acquired for the period of time from the start of operations to five months post The Summit’s opening. These data will be analyzed to understand penetration (e.g., proportion of patients filling out PROMs at planned timepoints, demographic and clinical characteristics of patients responding to PROMs) and appropriateness of measures (e.g., percentage of missing responses, associations between PROMs and with clinician-reported outcomes).

### Data analysis

*Qualitative data* Qualitative data sources include open-ended survey questions, interviews with clinical staff and focus group data. We will initially use a deductive, directed content analysis approach, as described by Hsieh and Shannon (2015), guided by CFIR, to analyze barriers and facilitators to implementation [39]. To provide a more in-depth description of patient and caregiver experiences with and perceptions of MBC, as well as clinical staff’s experiences (separately) a second analysis of qualitative data will be performed using inductive, conventional content analysis [39]. Qualitative data analysis will be performed using NVIVO qualitative data analysis software [40].

*Staff survey data* Descriptive statistics will be calculated for demographic and closed-response questions from the survey.

*De-identified EHR data* To specifically examine penetration of the intervention in The Summit, the percentage of patients participating in MBC (i.e., filling out PROMs) will be calculated using the total number of patients enrolled in the services compared with the number of patients who responded to PROMs. The percentage of patients filling out PROMs at intake, midpoint and discharge will also be calculated (as a measure of fidelity and penetration).

*PROMs data* The PROMs data of those who consented to research use will be used to perform subgroup analyses by service (i.e., walk-in, day program, intensive community treatment) and by patient demographics (e.g., age, sex, gender, spoken language, education level).

To examine the validity of the chosen PROMs for use in MBC in this population, analyses of item quality, construct validity and responsiveness (ability to capture change) will be performed on the PROMs data of those who consent for their data to be used. Item analysis will be performed by calculating descriptive statistics for the individual items in each PROM will be calculated (median of the responses, the frequency of endorsement, i.e., the proportion of people choosing each response option, and non-response rates i.e., percent of missing data). Construct validity of the PROMs will be evaluated using a hypothesis-testing approach recommended by the COSMIN group [41]. Responsiveness of the measures will be evaluated using Guyatt’s responsiveness ratio [42].

## Discussion

Once the initial implementation process and evaluation are complete, we will have assessed the progress towards the full implementation of MBC at The Summit, as well as identified factors that are affecting implementation. The findings will inform the co-design of strategies with key stakeholders (implementation team, clinical staff, patient and caregiver advisory councils) to address identified barriers and enhance facilitators, and potentially adapt components of the MBC intervention as well as the PROMs workflows.

Previous studies have described the implementation of PROMs within pediatric clinical care settings [43–46], or studied the effectiveness of MBC in child and youth mental health populations [22]. Two studies have evaluated aspects of implementation of MBC in child and youth mental health settings in the United States [47, 48]. To our knowledge, this is the first to study the implementation of PROMs in a publicly funded, Canadian child and youth mental health setting. A systematic evaluation of the implementation of MBC can identify barriers and facilitators, and successful strategies for implementing PROMs in clinical care for child and youth mental health setting. This information can be used to improve future implementation efforts in similar settings.

Foley and Vale (2022) described four key questions to ask during the development of an LHS: (1) what is the rationale for the LHS, (2) what sources of complexity exist in the system; (3) which strategic approaches to change need to be considered; (4) what technical building blocks are needed for a functioning LHS? [14]. The integration of PROMs is one of the aspects considered under the fourth question, the technical building blocks (i.e., collecting data from practice) for developing The Summit as part of an LHS. Other technical building blocks are generating generalizable knowledge from practice data (i.e., analyzing and interpreting data), applying knowledge to practice (e.g., making changes to services, adjusting treatments offered), and learning communities [14]. Learning communities are comprised of multiple stakeholders who come together to address common problems of interest within the LHS [49]. They are at the centre of an LHS, driving the learning and improvement cycles that are key to an effective LHS [14]. In parallel to the implementation of PROMs at The Summit, learning communities will need to be planned and initiatiated [49]. This will involve identifying individuals, developing community structure and governance, sources of funding, defining the problem(s) of interest for the community (e.g., clinical research questions, quality improvement problems), and assessing the data needs for the project [49]. The Summit, with its academic partnership, has many of these elements in place, however processes will need to be formalized, for example, data-sharing agreements and processes between the academic and health system partners need to be established. A structure for a learning community for problem(s) of interest internal to The Summit (e.g., quality improvement initiatives) will also need to be planned.

### Potential issues for this study

We foresee two aspects of this project to create challenges for this evaluation: (1) issues related to change at a newly opening facility, and (2) the research versus quality improvement paradigm that currently exists in health research taking place within health systems.

The Summit is a facility which has yet to begin offering services. Its clinical services were designed based on perceived needs of youth with mental health concerns in Calgary and its staff is completely new. As such, it is likely that The Summit’s operational processes, the services offered and the populations it serves will evolve rapidly in the early stages of opening in response to the needs of patients, to improve workflow efficiencies and as available resources change. This will have two potential effects on this project. The first is that our pre-implementation assessment is limited to a theoretical assessment of barriers and facilitators based on literature, as well as the working group and their professional networks’ experiences of implementing similar interventions in other settings. This evolving assessment could have had substantial impacts on the design of our implementation strategies. Second, the implementation evaluation analyses will have to take into account the changes to The Summit’s operations that are likely to happen in the first year of its operations. Both of these factors increase the complexity of the evaluation and will make it more challenging to accurately describe barriers and implementation outcomes. However, the rate of change at a new facility underscores the need for this formative evaluation, which will uncover and address unanticipated barriers to PROM implemenation.

The second challenge relates to the distinction between quality improvement projects and research. AHS considers academic research a distinct activity, with different requirements than quality improvement projects. The collaboration between the AHS operations team and the academic researchers is foundational to The Summit as an LHS; however, since this evaluation is led by the academic partners, access to data for the evaluation is controlled by research policies within AHS. This has the potential effect of reducing the amount and quality of data that can be used for the evaluation, particularly in terms of the MBC PROMs database, because patients will have to provide informed consent for the use of their PROMs data for this evaluation, and not all patients will consent to this. This will be a limitation to the rigor of this evaluation. Conversely, if this project were run by the health system team, it would be considered a quality improvement project, and while patients should still be consenting to have their data used in quality improvement projects, the data informing this evaluation might be more comprehensive. This problem will also affect other learning activities within The Summit, and has been identified as an issue that hinders learning within health care systems more broadly [11, 50].

## Conclusion

Establishing the components of an LHS is one path towards providing high-value services to meet the growing demand for child and adolescent mental health care. PROMs data, generated as part of clinical care, feed into the LHS and also are suggested as a specific intervention, MBC, to improve outcomes for patients with mental health concerns. However, the implementation of PROMs is challenging, and the literature to guide the process is child and youth mental health is limited. This study has the potential to make a meaningful contribution to the science of implementing PROMs in services for this population.

### Supplementary Information

Additional file 1.

Additional file 2.

Additional file 3.

## Data Availability

Not applicable.

## Abbreviations

AHS: Alberta health services
LHS: Learning health system
VBHC: Value-based health care
PROM: Patient-reported outcome measure
PedsQL: Pediatric Quality of Life Inventory Generic Core Scales
RCADS-25: Revised Children’s Anxiety and Depression Scale
C-SSRS: Columbia-Suicide Severity Rating Scale
EHR: Electronic health record
CGAS: Children’s Global Assessment Scale
QIF: Quality implementation framework
MBC: Measurement-based care
IRLM: Implementation research logic model
CFIR: Consolidated framework for implementation research

## References

[1] World Health Organization. Adolescent mental health: World Health Organization; 2021 [Available from: https://www.who.int/news-room/fact-sheets/detail/adolescent-mental-health#:~:text=Globally%2C%20one%20in%20seven%2010,illness%20and%20disability%20among%20adolescents.

[2] Solmi M, Radua J, Olivola M, Croce E, Soardo L, Salazar De Pablo G (2022). Age at onset of mental disorders worldwide: large-scale meta-analysis of 192 epidemiological studies. Mol Psychiatry.

[3] Statistics Canada. Table 13-10-0763-01 Health characteristics of children and youth aged 1 to 17 years, Canadian Health Survey on Children and Youth 2019. Statistics Canada; 2019.

[4] Barican JL, Yung D, Schwartz C, Zheng Y, Georgiades K, Waddell C (2022). Prevalence of childhood mental disorders in high-income countries: a systematic review and meta-analysis to inform policymaking. Evid Based Ment Health.

[5] Addictions and Mental Health—Interactive Health Data Application [Internet]. 2022. Available from: http://www.ahw.gov.ab.ca/IHDA_Retrieval/selectCategory.do?dataBean.id=302&command=doSelectSubCategory&cid=302.

[6] Callahan Soto E, Frederickson AM, Trivedi H, Le A, Eugene MC, Shekher M (2009). Frequency and correlates of inappropriate pediatric psychiatric emergency room visits. J Clin Psychiatry.

[7] Lee J (2021). Calgary kids and teens face growing mental health crisis as pandemic drags on. CBC News.

[8] Vaillancourt T, Szatmari P, Georgiades K, Krygsman A (2021). The impact of COVID-19 on the mental health of Canadian children and youth. FACETS.

[9] Statistics Canada. Table 98-10-0002-02 Population and dwelling counts: Canada, provinces and territories, and census subdivisions (municipalities). 2022.

[10] Porter ME (2010). What is value in health care?. N Engl J Med.

[11] Menear M, Blanchette M-A, Demers-Payette O, Roy D. A framework for value-creating learning health systems. Health Res Policy Syst. 2019;17(1). 10.1186/s12961-019-0477-3 PMC6688264 31399114

[12] Wasylak T, Benzies K, McNeil D, Zanoni P, Osiowy K, Mullie T (2023). Creating value through learning health systems: the alberta strategic clinical network experience. Nurs Adm Q.

[13] Canadian Institutes for Health Information. PROMS: Background document. Canadian Institute for Health Information. 2015:36.

[14] Foley T, Vale L (2022). A framework for understanding, designing, developing and evaluating learning health systems. Learning Health Syst.

[15] Ishaque S, Karnon J, Chen G, Nair R, Salter AB (2019). A systematic review of randomised controlled trials evaluating the use of patient-reported outcome measures (PROMs). Qual Life Res.

[16] Santana M-J, Feeny D (2014). Framework to assess the effects of using patient-reported outcome measures in chronic care management. Qual Life Res.

[17] Foster A, Croot L, Brazier J, Harris J, O’Cathain A. The facilitators and barriers to implementing patient reported outcome measures in organisations delivering health related services: a systematic review of reviews. J Patient-Reported Outcomes. 2018;2(1). 10.1186/s41687-018-0072-3 PMC6170512 30363333

[18] Santana MJ, Haverman L, Absolom K, Takeuchi E, Feeny D, Grootenhuis M (2015). Training clinicians in how to use patient-reported outcome measures in routine clinical practice. Qual Life Res.

[19] Austin EJ, Lerouge C, Lee JR, Segal C, Sangameswaran S, Heim J, et al. A learning health systems approach to integrating electronic patient‐reported outcomes across the health care organization. Learning Health Syst. 2021;5(4). 10.1002/lrh2.10263 PMC8512814 34667879

[20] Haverman L, Limperg PF, Young NL, Grootenhuis MA, Klaassen RJ (2017). Paediatric health-related quality of life: what is it and why should we measure it?. Arch Dis Child.

[21] McCabe E, Rabi S, Bele S, Zwicker JD, Santana MJ. Factors affecting implementation of patient-reported outcome and experience measures in a pediatric health system. J Patient Reported Outcomes under review. 10.1186/s41687-023-00563-1 PMC9998780 36892738

[22] Parikh A, Fristad MA, Axelson D, Krishna R (2020). Evidence base for measurement-based care in child and adolescent psychiatry. Child Adolesc Psychiatr Clin N Am.

[23] Matza LS, Swensen AR, Flood EM, Secnik K, Leidy NK (2004). Assessment of health-related quality of life in children: a review of conceptual, methodological, and regulatory issues. Value Health.

[24] Lewis CC, Boyd M, Puspitasari A, Navarro E, Howard J, Kassab H (2019). Implementing measurement-based care in behavioral health: a review. JAMA Psychiat.

[25] International Consortium for Health Outcomes Measurement. Children & Young People with Anxiety & Depression, Including OCD & PTSD: Data Collection Reference Guide. Boston, MA: International Consortium for Health Outcomes Measurement, 2022.

[26] Varni J, Seid M, Kurtin P (2001). PedsQL 4.0: reliability and validity of the Pediatric Quality of Life Inventory version 4.0 generic core scales in healthy and patient populations. Med Care.

[27] Ebesutani C, Reise S, Chorpita B, Ale C, Regan J, Young J (2012). The Revised Child Anxiety and Depression Scale-Short Version: scale reduction via exploratory bifactor modeling of the broad anxiety factor. Psychol Assess.

[28] Posner K, Brown GK, Stanley B, Brent DA, Yershova KV, Oquendo MA (2011). The columbia-suicide severity rating scale: initial validity and internal consistency findings from three multisite studies with adolescents and adults. Am J Psychiatry.

[29] Bele S (2022). Investigating the Implementation of Pediatric Patient-reported Outcome and Experience Measures in Alberta [Dissertation].

[30] Schaffer D, Gould MS, Brasic J, Ambrosini P, Fisher P, Bird H (1983). A Children’s Global Assessment Scale (CGAS). Arch Gen Psychiatry.

[31] Meyers DC, Durlak JA, Wandersman A (2012). The quality implementation framework: a synthesis of critical steps in the implementation process. Am J Community Psychol.

[32] Snyder CF, Aaronson NK, Choucair AK, Elliott TE, Greenhalgh J, Halyard MY (2012). Implementing patient-reported outcomes assessment in clinical practice: a review of the options and considerations. Qual Life Res.

[33] International Society for Quality of Life Research. User’s Guide to Implementing Patient-Reported Outcomes Assessment in Clinical Practice. In: (prepared by Aaronson N ET, Greenhalgh J, Halyard M, Hess R, Miller D, Reeve B, Santana M, Snyder C), editor. 2015. 10.1007/s11136-011-0054-x 22048932

[34] Smith JD, Li DH, Rafferty MR (2020). The Implementation Research Logic Model: a method for planning, executing, reporting, and synthesizing implementation projects. Implement Sci.

[35] Stetler CB, Legro MW, Wallace CM, Bowman C, Guihan M, Hagedorn H (2006). The role of formative evaluation in implementation research and the QUERI experience. J Gen Intern Med.

[36] Creswell J, Plano CV (2007). Designing and conducting mixed methods research.

[37] Damschroder LJ, Reardon CM, Widerquist MAO, Lowery J (2022). The updated Consolidated Framework for Implementation Research based on user feedback. Implement Sci.

[38] Chan AW, Tetzlaff JM, Altman DG, Laupacis A, Gotzsche PC, Krleza-Jeric K (2013). SPIRIT 2013 statement: defining standard protocol items for clinical trials. Ann Intern Med.

[39] Hsieh H-F, Shannon SE (2015). Three approaches to qualitative content analysis. Nordic J Digit Lit.

[40] Ltd QIP. NVivo. 12.5.0 ed: QRS International; 2018.

[41] Terwee CB, Bot SDM, de Boer MR, van der Windt DAWM, Knol DL, Dekker J (2007). Quality criteria were proposed for measurement properties of health status questionnaires. J Clin Epidemiol.

[42] Guyatt G, Walter S, Norman G (1987). Measuring change over time: assessing the usefulness of evaluative instruments. J Chronic Dis.

[43] Engelen V, Haverman L, Koopman H, Schouten van Meeteren N, Meijer van den Bergh E, Vrijmoet-Wiersma J (2010). Development and implementation of a patient reported outcome intervention (QLIC-ON PROfile) in clinical paediatric oncology practice. Patient Educ Couns.

[44] Schepers SA, Sint Nicolaas SM, Haverman L, Wensing M, Schouten van Meeteren AYN, Veening MA (2017). Real-world implementation of electronic patient-reported outcomes in outpatient pediatric cancer care. Psychooncology.

[45] Haverman L, van Oers HA, Limperg PF, Hijmans CT, Schepers SA, Sint Nicolaas SM (2014). Implementation of electronic patient reported outcomes in pediatric daily clinical practice: the KLIK experience. Clin Pract Pediatric Psychol.

[46] Bele S, Rabi S, Zhang M, Oddone Paolucci E, Johnson DW, Quan H, et al. Patient-reported outcome measures in pediatric asthma care: using theoretical domains framework to explore healthcare providers’ perceptions. J Patient-Reported Outcomes. 2022;6(1). 10.1186/s41687-022-00494-3 PMC9389517 35984533

[47] Kotte A, Hill KA, Mah AC, Korathu-Larson PA, Au JR, Izmirian S (2016). Facilitators and barriers of implementing a measurement feedback system in public youth mental health. Adm Policy Mental Health.

[48] Williams NJ, Marcus SC, Ehrhard MG, Sklar M, Esp SM, Carandang K, et al. Randomized Trial of an Organizational Implementation Strategy to Improve Measurement-Based Care Fidelity and Youth Outcomes in Community Mental Health. Journal of the American Academy of Child & Adolescent Psychiatry. 2023; Available online December 2023. 10.1016/j.jaac.2023.11.010 PMC11265517 38070868

[49] Ferguson L, Dibble M, Ferraro J, Williams M. Operationalizing a Learning Community for a Learning Health System: A Practical Guide. Department of Learning Health Sciences, University of Michigan Medical School; 2020.

[50] Faden RR, Kass NE, Goodman SN, Pronovost P, Tunis S, Beauchamp TL. An ethics framework for a learning health care system: a departure from traditional research ethics and clinical ethics. Hastings Cent Rep. 2013;Spec No:S16–27. 10.1002/hast.134 23315888

