# Supplementary figures and images for: A protocol for the formative evaluation of the implementation of patient-reported outcome measures in child and adolescent mental health services as part of a learning health system
Source: Health Res Policy Syst. 2024 Jul 15;22:85. doi: 10.1186/s12961-024-01174-y (PMC11251393; doi:10.1186/s12961-024-01174-y)

# Supplementary File 1: Implementation Research Logic Model (IRLM)

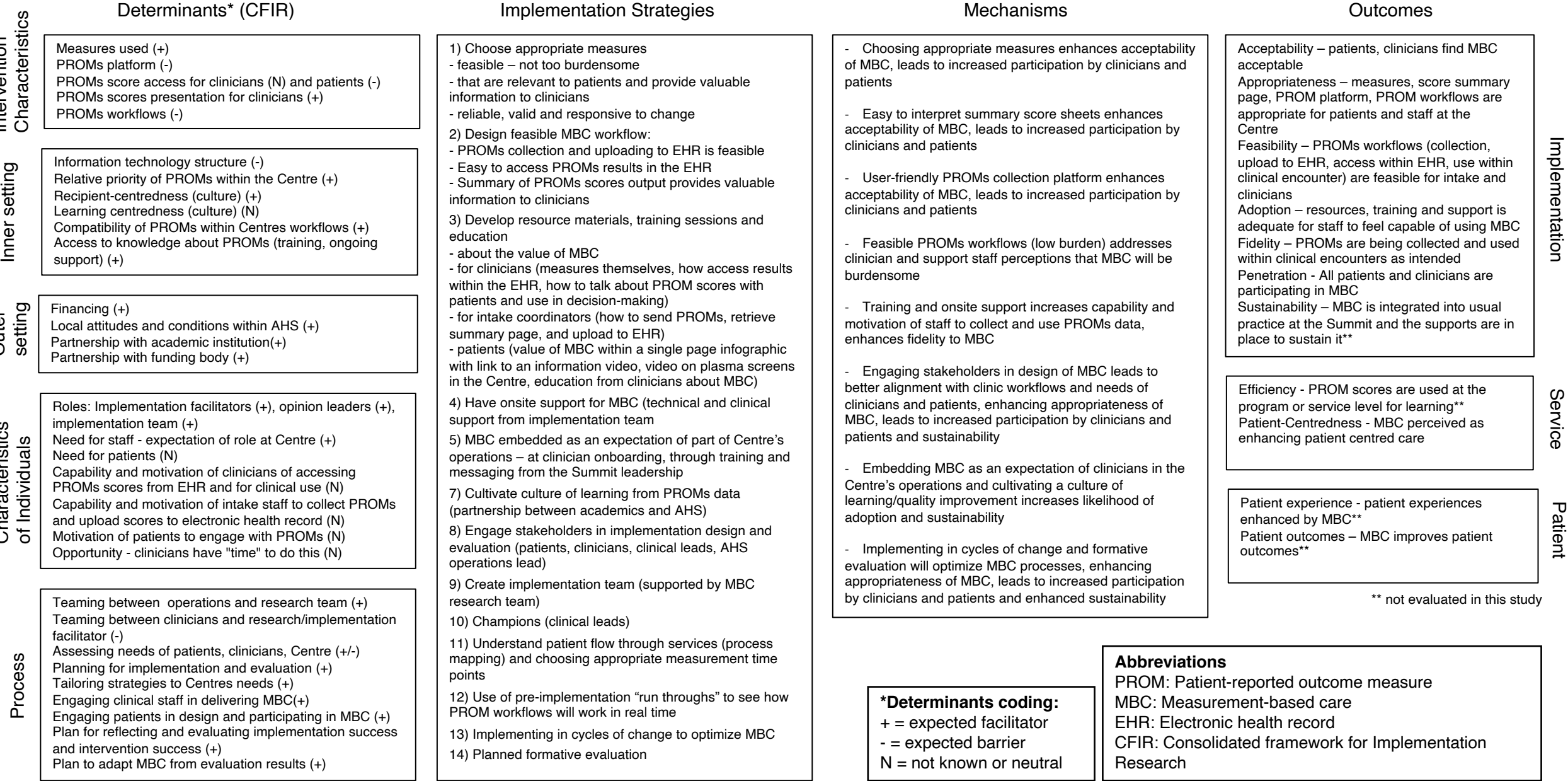

Supplement: Supplementary file 1 — Additional file 1. [file 12961_2024_1174_MOESM1_ESM.pdf]
