# Supplementary material for: A protocol for the formative evaluation of the implementation of patient-reported outcome measures in child and adolescent mental health services as part of a learning health system
Source: Health Res Policy Syst. 2024 Jul 15;22:85. doi: 10.1186/s12961-024-01174-y (PMC11251393; doi:10.1186/s12961-024-01174-y)
Supplement: Supplementary file 2 — Additional file 2. [file 12961_2024_1174_MOESM2_ESM.docx]

# Supplementary File 2: Evaluation Plan

# Intervention brief description

Measurement-based care (MBC) refers to the routine use of patient-reported outcome measures (PROM) to inform mental health treatment. MBC consists of three main steps: (1) administering a symptom, outcome, or process measure (i.e., PROM) being administered (ideally before a clinical encounter); (2) clinician and patient review of PROM data; (3) collaborative re-evaluation of the treatment plan informed by data.

MBC is part of the standard of care at the Summit. Three PROMs will be used, the acute version of the Pediatric Quality of Life Inventory Generic Core Scales (PedsQL), the Revised Children's Anxiety and Depression Scale (RCADS-25), and Columbia-Suicide Severity Rating Scale (C-SSRS). For walk-in services, one PROM (PedsQL) will be administered routinely at intake, with an intake questionnaire flagging whether RCADS-25 and C-SSRS need to be used. For the 2 week-long day program, a step down program after an inpatient psychiatric admission, 3 PROMs will be administered at intake and at discharge. For intensive community treatment services, which are designed to provide short-term (5-6 weeks), intensive support to children and youth in crisis, 3 PROMs are administered at intake, midpoint of treatment and at discharge. In each case, the PROMs will be automatically scored and represented graphically (along with prior PROM scores) in a downloadable document which also contains participants’ responses to each item. The document will be imported into the patient’s electronic health record. During the clinical encounter, the clinician will access patient scores and review them with the patient. Together they will discuss patient concerns highlighted by the PROM and re-evaluate the treatment plan.

# Stakeholders

1. Scientific– Measurement-based care subcommittee and patient-oriented research and evaluation team
2. Health system leaders – The Summit Operations Manager, Patient and Family-Centred Care Coordinator, Clinical Leads
3. Clinical staff (mental health therapists, psychiatrists, clinical support staff)
4. Patients (youth, caregivers)
5. Alberta Health Services Provincial Addictions and Mental Health

# Primary outcomes

1. Appropriateness
2. Penetration
3. Fidelity

# Evaluation objectives

1. Assess the implementation progress to date (penetration, fidelity, appropriateness and adoption)
2. Identify barriers and facilitators to implementation and sustainability of PROMs at the Summit
3. Explore patient and clinician experiences of PROMs in clinical care

# Evaluation Plan

| **Implementation outcomes** | **Definition** | **Evaluation questions** | **Indicators** | **Data sources** |
| --- | --- | --- | --- | --- |
| Acceptability | The perception among implementation stakeholders that a given treatment, service, practice, or innovation is agreeable, palatable, or satisfactory. | - Is the use of PROM in clinical practice perceived as beneficial to patients and clinicians? - What are the attitudes towards PROMs in clinical care? - Is making responses “required” an issue? | - Patients/clinicians find MBC acceptable | Interviews/focus groups with patients, clinicians  Surveys for clinicians |
| Appropriateness | The perceived fit, relevance, or compatibility of the innovation or evidence based practice for a given practice setting, provider, or consumer; and/or perceived fit of the innovation to address a particular issue or problem. | - Are the chosen PROMs the best fit for the Centre’s 3 services? - Is the PROMs platform appropriate for the services? - Are the timepoints for measurement appropriate? - Is the summary score page useful for clinicians/patients? - How are clinicians using parent vs self-reports? | - PROMs fit to patients (relevant to symptoms, easy to understand) - PROMs fit to clinicians (relevant to patient’s symptoms, easy to interpret, add valuable information to encounters, useful for decision-making) - Measurement frequency is appropriate - Summary score page is useful/enhances information from PROMs scores - PROMs collection platform is easy to use (for patients) - Validity of measures for their use – relationships between measure scores and domain scores, including clinician reported measure, testing responsiveness (are they capturing change), testing MCID - % Missing responses in each measure (correlated with demographics/service) | Interviews/focus groups with patients, clinicians  All patients’ MBC PROMs data consenting for research use |
| Feasibility | The extent to which a new treatment, or an innovation, can be successfully used or carried out within a given agency or setting. | - Are the PROMs workflows perceived as feasible to intake staff and clinicians? - Is using measures in discussion with patients feasible? - Is collecting PROMs at a time point after discharge feasible? | - Admin, intake and clinicians say PROM workflows are feasible - PROMs collection and upload to EMR is feasible - PROMs are accessible within the EMR for clinicians | Interviews/focus groups with patients, clinicians, leaders, support staff |
| Adoption |  | - Is training adequate/useful for implementing PROMs? - Is the onsite support for PROMs adequate/useful? - Are resource guide and videos adequate to initiate PROMs use/implementation? - -Does leadership/management seem to value MBC? | - Clinicians feel capable of using MBC as part of clinical care - Clinicians feel that MBC is of value and a priority for management | Interviews/focus groups with patients, clinicians |
| Fidelity | The degree to which an intervention was implemented as it was prescribed in the original protocol or as it was intended by the program developers. | - Are PROMs data being collected and uploaded as intended? - Are PROMs scores being used as intended? | - Proportion of providers charting on the review and discussion of the PROMs within the encounter - Proportion of patient-visits where REDcap MBC data is uploaded to their EMR - Providers and patients reporting use of PROMs as intended | Chart review of random sample of charts  Interviews with patients, clinicians |
| Penetration/Reach | The integration of a practice within a service setting and its subsystems. | - To what extent are PROMs being completed and used as intended within each of 3 services and by clinicians? - What types of patients (demographics, service types) are/are not completing PROMs? (equity) | - Absolute # and proportion of patients filling out each of PROMs at planned time points - Representativeness - proportion of patients with PROMs data per clinician by clinician role, specialty, gender - Comparison of characteristics of patients who complete vs don’t complete - Absolute # and proportion of clinicians/managers attending training sessions | All patients’ MBC PROMs data consenting for research use |
| Sustainability | The extent to which a newly implemented treatment is maintained or institutionalized within a service setting’s ongoing, stable operation. | - What are the barriers and facilitators to sustaining use of PROMs? - What support is required to sustain the use of PROMs in clinical care? | - PROMs use is sustained over 1 year by 80% of clinicians | Interviews/focus groups with patients, clinicians, managers |

| **Service outcomes** | **Definition** | **Evaluation questions** | **Indicators** | **Data sources** |
| --- | --- | --- | --- | --- |
| Efficiency | Avoiding waste, including waste of equipment, supplies, ideas, and energy. | - Are PROMs data being used as a source for quality improvement/learning within the healthcare system? | There is a system in place for using PROMs data for learning in the Summit | Not evaluated |
| Patient-centeredness | Providing care that is respectful of and responsive to individual patient preferences, needs, and values and ensuring that patient values guide all clinical decisions. | - Does MBC enhance the patient experience of patient- and family-centred care? | Patients perceive MBC as a mode of increasing patient-centredness of services. | Focus groups with patients |

| **Clinical outcomes** | **Evaluation questions** | **Indicators** | **Data sources** |
| --- | --- | --- | --- |
| Patient experience | - Does MBC enhance patient’s experience of feeling involved in treatment decision making? | Patients experiences at the Summit are positively impacted by MBC | Not evaluated |
| Patient outcomes | - Does MBC result in improved clinical outcomes? | Patient outcomes at the Summit are positively impacted by MBC. | Not evaluated |
